# Effect of vaginal antiseptic prior to caesarean section on the rate of post-caesarean complications: a blinded randomised controlled trial

**DOI:** 10.1186/s13063-021-05857-7

**Published:** 2022-03-24

**Authors:** Monika Trivedi, Ainsley M. Robinson, Md Rafiqul Islam

**Affiliations:** 1 Goulburn Valley Health, Graham Street, Shepparton, Victoria 3630 Australia; 2 Department of Rural Health, The University of Melbourne, Shepparton, Victoria 3630 Australia; 3 Rural Health School, La Trobe University, Shepparton, Victoria 3630 Australia

**Keywords:** Caesarean section, Vaginal cleansing, Antiseptic wash, Post-caesarean infection

## Abstract

**Background:**

Rates of caesarean section (CS) delivery are increasing worldwide. CS delivery is often complicated by post-surgical infection, estimated to have ten times higher risk of infections than that of vaginal delivery. While widespread use of prophylactic antibiotics with CS has reduced post-CS infection, incidence may be reduced further by cleansing the vagina with betadine antiseptic wash prior to CS. However, reports are not consistent, and different antiseptics have been practised variably. Therefore, in order to ensure that the risks to the mother are as minimal as possible, it is important to determine whether vaginal irrigation with antiseptic wash reduces post-CS infection rate, and if so, which antiseptic is paramount.

**Methods:**

Women giving birth by elective or emergency CS will be assigned into either the intervention (1% povidone iodine (*n* = 125) or chlorhexidine (*n* = 125)) or the control (no-irrigation (*n* = 125)) group by using a block randomisation technique. Participants will receive vaginal cleansing with an intervention or no vaginal cleansing prior to CS. Follow-up will occur at day 14 and day 28 post-CS. A predeveloped questionnaire will be completed with patients’ socio-demographic characteristics and required clinical and pregnancy-related information. All the fever, infection and readmission-related information will be completed from either the patient’s or their record or at follow-up visits. Occurrence of post-CS infection, as measured by primary and secondary outcomes, will be compared between the groups.

**Discussion:**

The results of this study may provide important data to define the future uniform use of vaginal antiseptic wash immediately prior to CS and to determine the best antiseptic wash details in reducing post-operative infections or complications.

**Trial registration:**

Australian New Zealand Clinical Trials Registry (ANZCTR) ACTRN12620000971932p. Registered on 28 September 2020

## Administrative information

Note: the numbers in curly brackets in this protocol refer to SPIRIT checklist item numbers. The order of the items has been modified to group similar items (see http://www.equator-network.org/reporting-guidelines/spirit-2013-statement-defining-standard-protocol-items-for-clinical-trials/).

**Table:** 

Title {1}	Effect of vaginal antiseptic prior to caesarean section on the rate of post caesarean complications: A blinded randomised controlled trial
Trial registration {2a and 2b}.	Trial identifier: ACTRN12620000971932p Registry name: Australian New Zealand Clinical Trials Registry (ANZCTR)
Protocol version {3}	Version 6, dated 18 December 2020.
Funding {4}	This study is unfunded, but internally sponsored by the Department of Obstetrics and Gynaecology, Goulburn Valley Health.
Author details {5a}	Monika Trivedi^1^*, Ainsley M Robinson^1^, Md Rafiqul Islam^1,2,3^ ^1^Goulburn Valley Health, Graham Street, Shepparton, Victoria 3630, Australia ^2^Department of Rural Health, The University of Melbourne, Shepparton, Victoria 3630, Australia ^3^Rural Health School, La Trobe University, Shepparton, Victoria 3630, Australia *Corresponding author
Name and contact information for the trial sponsor {5b}	Department of Obstetrics and Gynaecology Goulburn Valley Health Graham Street Shepparton, Victoria, 3630 Phone: 03 5823 8670
Role of sponsor {5c}	There are no sources of funding for this study. M.T. is employee of the sponsor, Goulburn Valley Health Obstetrics and Gynaecology Department, and along with MRI were/will be involved in the study design; collection, management, analysis, and interpretation of the data; in the writing of this manuscript; and in the decision to submit the article for publication along with all above-mentioned support from the other author AMR as well as support and guidance from MRI of the Research and Ethics Department, Goulburn Valley Health.

## Introduction

### Background and rationale {6a}

Caesarean section (CS) is one of the methods of operative delivery in childbirth, and its rate has increased internationally over the last 3 decades for various reasons. According to the Australian Institute of Health and Welfare, the CS rate in Australia was 34% in 2016 [1]. Similar to any other surgical procedure, CS has morbidity in the form of infections, which include endometritis in addition to surgical site infection (SSI). This is a matter of concern as it has been reported that CS can have ten times higher risk of infections than that of vaginal delivery [2]. Furthermore, higher rates of infection have been identified in women with ruptured membranes and in women who undergo a CS in labour [3]. Such infections cause additional burden not only to the mother herself, but also to the newborn and her family as a whole. It also increases the risk of hospital readmissions and cost to health care systems across the world [4]. Olsen et al. demonstrated that wound infection and endometritis may incur an additional cost of US $4200 and US $4500 to the health systems, respectively [5]. Evidence favours the use of prophylactic antibiotics for CS to reduce post-operative infections by 60–70%, as well as the cost and duration of hospitalisation [6–8]. However, there are debates regarding single versus multiple doses, routes and timing of antibiotic use, type of antibiotics and duration. To reduce the rate of post-CS infections further, different antiseptics have been practised inconsistently for skin preparation and vaginal toileting. It has been demonstrated that vaginal preparation with povidone iodine or chlorhexidine solution compared to saline or not cleansing immediately before CS probably reduces the risk of post-CS infection [3]. However, a Cochrane review suggests no clear evidence favouring the use of chlorhexidine solution before surgery over other washing products to prevent SSI [9], while another Cochrane review reported that use of either chlorhexidine or povidone iodine before CS did not make any or might make little difference to the SSI or endometritis [2]. On the other hand, the World Health Organization (WHO) recommends the use of povidone iodine for vaginal cleansing immediately before CS to reduce maternal infection morbidities [10]. This recommendation is further emphasised in a recent systematic review and network meta-analysis by Roeckner et al. which reported that the use of 1% povidone iodine for pre-surgical vaginal irrigation among women who underwent CS had the most beneficial outcomes in the reduction of fever, wound infections and endometritis [11]. Despite the above measures, SSI incidence ranges from 3 to 15% [12], perhaps due to the variability of practices and individual preferences to one antiseptic agent over the other. Given the current limited data, it is imperative to identify the best evidence-based practice for uniform use of antiseptic agents for vaginal toileting to reduce post-CS infections or complications and to standardise their practice in a regional Australian hospital for enhanced recovery after caesarean delivery (ERAs).

### Objectives {7}

#### Hypothesis

H_1_: In a blinded randomised controlled clinical trial, using either chlorhexidine or povidone iodine for vaginal irrigation prior to CS will reduce post-operative infection outcomes by 50% when compared to no vaginal irrigation.

#### Aims

The aims are to introduce uniform use of vaginal antiseptic wash immediately prior to CS and to determine the best antiseptic wash material in reducing post-operative infections or complications.

#### Objectives

The following are the objectives:

1. To determine the number of post-CS infections among women that will undergo pre-operative vaginal cleansing with either 1% povidone iodine or chlorhexidine or no toileting (control) during elective CS
2. To determine the number of post-CS infections among women that will undergo pre-operative vaginal cleansing with 1% povidone iodine or chlorhexidine or no toileting (control) during emergency CS
3. To compare the rate of infections among the three groups that receive either 1% povidone iodine or chlorhexidine or no toileting for vaginal irrigation prior to any kind of CS
4. To identify the best antiseptic wash and methods for vaginal irrigation prior to CS for developing a uniform guideline through locally generated evidence
5. To determine the factors that may influence the outcomes, such as post-CS infections or related complications

### Trial design {8}

This will be a single-centre, three-arm, single-blind, randomised controlled trial. A total of 396 eligible patients will be randomised in a 1:1:1 ratio to receive pre-operative vaginal cleansing with 1% povidone iodine or chlorhexidine or no toileting (control) immediately prior to CS.

## Methods: participants, interventions and outcomes

### Study setting {9}

The study will be conducted in the Obstetrics and Gynaecology Department of Goulburn Valley Health, a regional Australian public hospital.

### Eligibility criteria {10}

#### Inclusion criteria

The inclusion criteria are any patients undergoing either emergency or elective CS that provide voluntary informed consent.

#### Exclusion criteria

The following are the exclusion criteria:

- Patients having signs of chorioamnionitis, intrapartum pyrexia and other signs of infection will be excluded from the study.
- Patients who develop intrapartum pyrexia as a result of syntocinon drip, prolonged labour or obstructed labour and provided voluntary informed consent will be included in the study initially. Their placental swabs will be taken for microscopic examination, culture and sensitivity (mcs). They will be excluded from the study only if their swab results are positive for mcs.
- Face presentation.
- Vaginal delivery.
- Patients who have known allergies to iodine or chlorhexidine.

#### Dropout criteria

The following are the dropout criteria:

- Patients who withdraw their consent during the study
- Patients who cannot be contacted at all for follow-up

### Who will take informed consent? {26a}

Informed consent will be obtained by the principal investigator or their sub-investigators.

Although this study focuses on delivery by CS (both elective and emergency) and not by the vagina, we are inviting all expecting pregnant women attending Goulburn Valley Health Obstetrics and Gynaecology Department to participate, since emergency CS delivery with intended vaginal delivery is mostly unpredictable. Patients will be approached to participate in the study by one of the sub-investigators or the principal investigator from the treating team. The investigator will be responsible for providing each patient with a participant information and consent form (PICF) about the study’s purpose and procedures, foreseeable benefits and potential risks of participation, information on data protection procedures and option to withdraw from the study at any time and without any given reason, which should be read by the patient. The investigator will answer any questions the patient may have, and both the patient and investigator will sign the informed consent form to indicate the patient’s full understanding of the protocol. Written informed consent must be obtained for all participants prior to any trial-related procedures and is subject to prior confirmation that the inclusion/exclusion criteria during the eligibility assessment are met for the enrolment of the patient.

### Additional consent provisions for collection and use of participant data and biological specimens {26b}

In the PICF, the participants will be informed about the use and storage of personal data collected during their participation in the trial. The PICF also contains information concerning the personnel who can access personal data collected during this trial and the period that the data will be kept following the study’s completion. By signing the informed consent form, the participants agree to the terms addressed in the PICF.

This trial does not involve any collection or storage of biological specimens.

### Interventions

#### Explanation for the choice of comparators {6b}

Different antiseptics or cleansing without antiseptic have been practised inconsistently, and there is no clear evidence supporting which vaginally administered antiseptic solution is the most effective for preventing post-CS infections. The choice of comparators is based on the common variations of antiseptic solutions for vaginal preparation before CS. No toileting was chosen as a control to best determine any effects of the interventions.

#### Intervention description {11a}

Patients undergoing elective or emergency CS are randomised to receive either of the following:

1. 1% povidone iodine antiseptic solution for vaginal cleansing immediately prior to CS (intervention)
2. Chlorhexidine antiseptic solution (aqueous) for vaginal cleansing immediately prior to CS (intervention)
3. No vaginal cleansing immediately prior to CS (control)

All the enrolled patients’ skin preparation will follow the same procedure and use of the same material of similar quantity (chlorhexidine). All enrolled patients will receive the same health education and wound care advice from the treating team.

#### Criteria for discontinuing or modifying allocated interventions {11b}

If, for any reason, a study intervention is not administered at the scheduled time, immediately prior to CS, it may not be administered later, and the patient will be withdrawn from the study. A patient may also be withdrawn from the study for the following reasons: (1) development of intrapartum pyrexia as a result of syntocinon drip, prolonged labour or obstructed labour intrapartum pyrexia and positive m/cs swab test; (2) withdrawal of consent from the study; (3) lost to follow-up; or (4) vaginal delivery is successful and emergency CS is not necessitated.

#### Strategies to improve adherence to interventions {11c}

The following measures will be, and or have been, taken to improve adherence to interventions and follow-up:

1. All participants will be informed of the study procedures, as well as potential benefits and risks to make them fully understand the significance of their involvement in the study.
2. Inform the participants about the detail of the follow-up period which is relatively short, 28 days follow-up from the day of CS.
3. Provide adequate details to the participants about the low or limited burden and inconvenience of data collection.

#### Relevant concomitant care permitted or prohibited during the trial {11d}

There will be no restrictions regarding concomitant care during the trial.

#### Provisions for post-trial care {30}

All patients will return to standard care after the trial. All the patients included in the study will be followed up for a period of 28 days for the outcome signs and symptoms. We will follow up the patients telephonically on the 14th and 28th post-operative days and also during any hospital presentations or admissions until 28 days post-CS.

### Outcomes {12}

#### Primary outcome

The primary outcome is endometritis: defined as tympanic temperature > 38 °C with uterine tenderness and/or foul-smelling vaginal discharge within 28 days of post-CS.

#### Secondary outcomes

The following are the secondary outcomes:

- Post-operative fever: defined as tympanic temperature > 38 °C at any point until 28 days post-CS
- Wound infection: defined as itch, redness, pain, swelling and collection of purulent discharge at the surgical incision site within 28 days post-CS
- Readmission with infection: defined as the representation of post-CS patients to the hospital within 28 days post-CS with surgical site infection or related infective complications

#### Assessment of infection

A third person with adequate clinical acumen independent to the project will assess the post-CS infection to reduce any potential bias, for example, the obstetrics and gynaecology consultant on call and obstetrics and gynaecology registrar.

### Participant timeline {13}

**Fig. 1:**
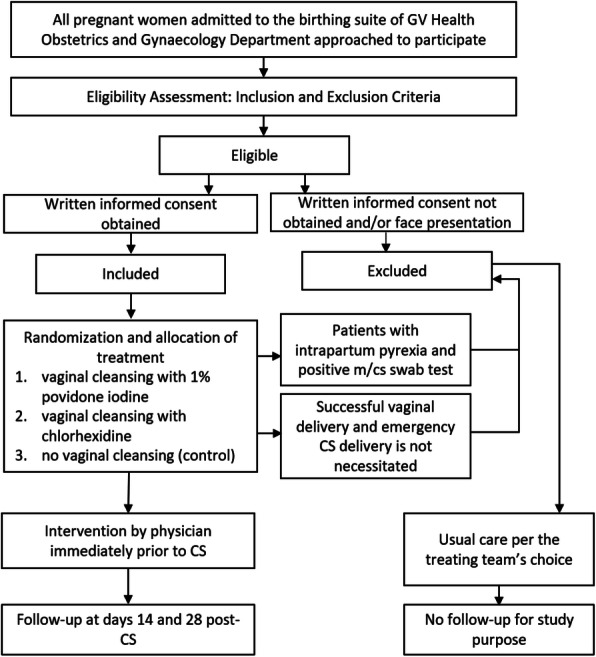
Flow chart demonstrating participant enrolment, randomisation, intervention and follow-up schedule

**Fig. 2:**
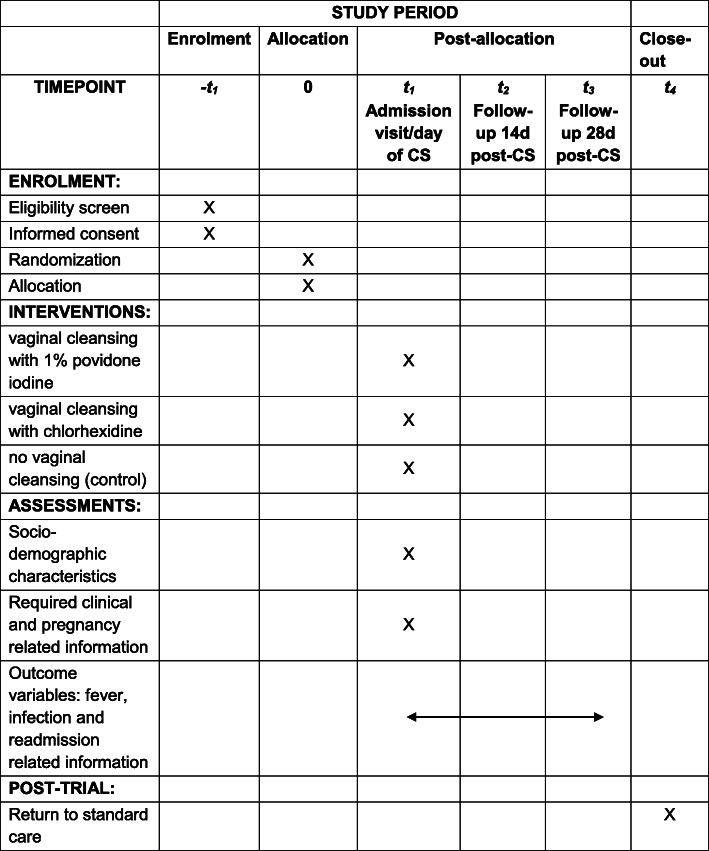
Standard Protocol Items: Recommendations for Interventional Trials (SPIRIT) table of enrolment, intervention and assessments

The study flowchart and participant timeline are presented in Figs. 1 and 2, respectively.

### Sample size {14}

We assume that the interventions will improve the rate of infection including post-operative infective complications (within 28 days post-CS) by 50% compared to the control. Thus, for sample size estimation in this study, we set *P*_1_ = 8% (as per overall prevalence of post-CS infection range 3–15%), *P*_2_ = 4%, significance level = 5% and the study power = 80%.

Therefore,

$$ n\ge {\left(\frac{Z_{\alpha }+{Z}_{\beta }}{\mathrm{ES}}\right)}^2 $$

$$ \mathrm{ES}=\frac{P_1-{P}_2}{\sqrt{P_1\left(1-{P}_1\right)}} $$

*n* ≥ 361

Here, *Z*_*α*_ = 1.96, *Z*_*β*_ = 0.84, *P*_1_ = 0.08, *P*_2_ = 0.04 and *P*_1_ − *P*_2_ = 0.04.

Considering 10% dropout, the study will require recruiting approximately 396 participants for all the groups (*n* = 132 in each group) over a period of 16 months to adequately test the assumption.

### Recruitment {15}

Participants will be recruited from the clinical setting. All expecting pregnant women attending Goulburn Valley Health Obstetrics and Gynaecology Department will be viewed as potential participants and invited to take part in the study; although this study concentrates purely on CS delivery (both elective and emergency) and not vaginal delivery, emergency CS delivery with intended vaginal delivery is mostly unpredictable. Considering CS deliveries comprise 25% of a total of approximately 1200 deliveries annually at Goulburn Valley Health, it is expected that we will recruit the required number of participants to reach the target sample size in 16-month period. Strategies to achieve adequate participant recruitment include the invitation of all pregnant women to participate, as well as study awareness and familiarity among departmental staff.

## Assignment of interventions: allocation

### Sequence generation {16a}

After inclusion in the trial, each prospective CS patient will be assigned either into the intervention (1% povidone iodine or chlorhexidine) or control (no-irrigation) group by using a block randomisation technique. A permuted block of six will be used to randomise the patients. A third party not related to the study will generate the permuted blocks and allocate the treatment in sealed opaque envelopes.

### Concealment mechanism {16b}

Opaque sealed envelopes will be made by a third party not related to the study and stored at the Goulburn Valley Health Obstetrics and Gynaecology Department. The envelopes will be not accessible to individuals directly involved in the study.

### Implementation {16c}

A third party, not involved in the assignment or care of the trial participants, will generate the permuted blocks for randomisation and place the assigned treatments in the sealed envelopes. Study investigators will lead participant recruitment, informing participants of all the trial procedures and obtaining their informed consent before the initial inclusion in the study. Study investigators will confirm the eligibility of the participant prior to any study procedures being undertaken. Prior to CS delivery, participants will pick one of the opaque sealed envelopes at random and pass it on to the treating team. Upon opening of the envelope, which designates chlorhexidine or 1% povidone iodine or no vaginal cleansing, the treating team will administer the allocation immediately prior to CS.

## Assignment of interventions: blinding

### Who will be blinded {17a}

The participants will remain blinded to the interventions.

### Procedure for unblinding if needed {17b}

There should not be any need to unblind the participants. The allocated treatment in each arm is commonly utilised within standard practice, and the intervention materials are well known to the treating team. Nevertheless, if required, unblinding can be carried out by the study site investigators.

## Data collection and management

### Plans for assessment and collection of outcomes {18a}

A predeveloped questionnaire will be completed with patients’ socio-demographic characteristics and required clinical and pregnancy-related information. All the fever, infection and readmission-related information such as duration of artificial rupture of membrane/spontaneous rupture of membrane, duration of labour, BMI, gestational diabetes mellitus (GDM) with current diet or insulin regime, duration of surgery, estimated blood loss (EBL), previous CS (1/2/3) of elective/emergency nature and vaginal examination at CS will be completed from either the patients or their record or at follow-up visits on the 14th and 28th post-operative days. All the patients included in the study will be followed up for a period of 28 days for the outcome signs and symptoms. Participants will be followed up over the phone fortnightly, at day 14 and day 28 post-CS. Participants will also be followed up during hospital presentations or admissions until 28 days post-CS. A third person with adequate clinical acumen independent to the project will assess the post-CS infection (primary and secondary outcomes) to reduce any potential bias, for example, the obstetrics and gynaecology consultant on call and obstetrics and gynaecology registrar. No identifying information but the aggregated data will be used for the analysis and interpretation.

### Plans to promote participant retention and complete follow-up {18b}

During participant recruitment, the purpose and importance of the trial will be explained via the study team and PICF. The participant has the right to withdraw from the trial at any time and for any reason without prejudice to her medical care and routine treatment by the treating team or at the institution. If a participant withdraws from the trial before concluding the study and since the data will be recorded anonymously, an intention-to-treat analysis will be performed for such patients’ data. If a patient is withdrawing their consent during the conduction of the trial and unwilling to consent for utilising already collected data and if these specific data can be retrieved, the investigator will delete the data accordingly.

### Data management {19}

Data will be collected on a paper questionnaire. The collected information will remain anonymous; participants will be allocated a participant number for de-identification purposes. The hard copy questionnaires will be scanned to create an electronic copy and the data transferred to a MS Excel database for assessment; data will be kept securely within the organisation’s network. All data will be stored with the principal investigator in a Goulburn Valley Health password-protected computer for a period of 5 years. Any hard copies of the data will be shredded/destroyed and disposed of in Goulburn Valley Health’s confidential paper/document collection bins and all soft copies of the data will be permanently deleted from the computer once the data storage time is over or at the conclusion of the study.

### Confidentiality {27}

Study investigators will ensure that the participants’ anonymity is maintained. Participant numbers are allocated for de-identification purposes; no identifiable information will be used but an aggregated data for dissemination. Prior to study participation, patients are informed that except for the study investigators, nobody will have access to the dataset unless there is an institutional or regulatory requirement. Hard copies of data are shredded/destroyed in confidential paper/document collection bins as soon as soft copies are made during the study. Soft copies are stored securely with the principal investigator on an organisational password-protected computer/network and will be destroyed 5 years after the study’s conclusion.

### Plans for collection, laboratory evaluation and storage of biological specimens for genetic or molecular analysis in this trial/future use {33}

No biological specimens will be collected for future genetic or molecular analysis.

## Statistical methods

### Statistical methods for primary and secondary outcomes {20a}

Baseline characteristics of participants including demographic characteristics, morbidity information with severity, current medication/management and satisfaction towards the current management will be compared using the *t*-test or and *χ*^2^ tests for categorical variables to assess the success of the randomisation. Summary statistics will be presented for all three groups’ infection information. BMI will be calculated using the weight and height of the participants. If required, the analysis will be performed following the ‘intention-to-treat’ analysis method. Primary and secondary outcomes will be compared using ANOVA. Where appropriate, logistic regression analyses will be used to compare the outcomes separately for interventions versus the control group. All data will be entered in MS Excel, and all statistical analyses will be performed using the STATA 11.0 statistical package.

### Interim analyses {21b}

No interim analyses are planned. It is unlikely that the trial will be stopped prematurely; however, the study can be temporarily suspended or prematurely discontinued for reasons such as safety and ethical concerns. The sponsoring organisation, relevant ethics committee and the research governance officer of the participating organisation authorities will be informed promptly of the decision including the reasons for such decision.

### Methods for additional analyses (e.g. subgroup analyses) {20b}

No additional analyses are planned.

### Methods in analysis to handle protocol non-adherence and any statistical methods to handle missing data {20c}

No imputation of missing data will be performed for statistical analysis.

### Plans to give access to the full protocol, participant-level data and statistical code {31c}

The full trial protocol will be shared on reasonable request. Anonymised data on the group level may be shared with outside investigators at the end of the trial after receiving institutional approval for releasing the data externally. The findings of this trial will be published in peer-reviewed journals and presented at conferences. The results of the study will be released to the participating patients.

## Oversight and monitoring

### Composition of the coordinating centre and trial steering committee {5d}

The trial will be conducted by the study research team, including the Goulburn Valley Health Obstetrics and Gynaecology Department and Goulburn Valley Health Research and Ethics Unit, in collaboration with the Obstetrics and Gynaecology Department staff. The principal investigator takes responsibility for supervision of the trial and ensures compliance with the study protocol. Vaginal cleansing prior to CS will be performed by the obstetrics and gynaecology study team clinicians. A third person with adequate clinical acumen independent to the project will assess the post-CS infection to reduce any potential bias, for example, the obstetrics and gynaecology consultant on call and obstetrics and gynaecology registrar. The statistical research plan and statistical analysis will be supervised by the Research and Ethics Unit, Goulburn Valley Health.

### Composition of the data monitoring committee, its role and reporting structure {21a}

The data monitoring committee is unnecessary in this trial, because this study will not involve participants with severe diseases and communication disabilities or interventions that can risk participants’ lives. Instead, the oversight of data quality will be provided by the Research and Ethics Unit, Goulburn Valley Health.

### Adverse event reporting and harms {22}

Since the proposed interventions in this study are safe and commonly used for vaginal cleansing prior to CS, no severe adverse events relating to the interventions are expected after careful enrolment following the specified exclusion criteria. However, all study patients will be monitored with respect to any possible adverse events related to the administration of interventions. As such, all adverse events (AEs) observed or reported by the patient are collected and evaluated for relatedness to trial intervention, seriousness, severity, expectedness and outcome. AEs are defined in the Good Clinical Practice (GCP) guideline.

### Frequency and plans for auditing trial conduct {23}

For the quality assurance, 5% randomly selected questionnaire from the collected information will be re-interviewed. Any discrepancies will be corrected accordingly. In addition, the study team will audit the data regularly. An audit may be performed by Goulburn Valley Health Research and Ethics Unit, its designee or regulatory agencies to evaluate the clinical study conduct and compliance with the protocol, standard operating procedures, GCP and the applicable regulatory requirements.

### Plans for communicating important protocol amendments to relevant parties (e.g. trial participants, ethical committees) {25}

The principal investigator will be responsible for any protocol amendments and their subsequent processes. Protocol amendments will be submitted to the Ethics Committee and implemented only after approval is received. It will be the responsibility of the principal investigator to disseminate the changes to the protocol, and all study research team members will be adequately trained on protocol amendments.

### Dissemination plans {31a}

The study findings will be presented internally in the Goulburn Valley Health Hospital for service improvement, externally in national or international conferences. A full scientific article will be developed and published in peer-reviewed journal/s. The results of the study will be released to the participating patients.

## Discussion

Post-operative infectious morbidity still complicates CS deliveries despite the widespread use of prophylactic antibiotics [3]. Although there is evidence suggesting that vaginal cleansing with antiseptic wash before CS reduces infection rates, different antiseptics have been practised inconsistently and there is no standard of practice for the uniform use of antiseptic agents, or a particular antiseptic agent, for vaginal toileting to reduce post-CS infections or complication. In this blinded randomised controlled clinical trial, we will evaluate the use of antiseptic wash for vaginal cleansing prior to CS and aim to determine the best antiseptic wash and methods to reduce post-CS infection. We hypothesise that the interventions, chlorhexidine or povidone iodine, will improve the rate of infection including post-operative infective complications within 28 days of CS by 50% compared to no vaginal irrigation. To test our hypothesis and meet the study aims and objectives, we will recruit 375 pregnant women giving birth by CS (*n* = 125 per group) over a 16-month period. The primary and secondary outcomes will be measured over 28 days post-CS to enable the determination of study conclusions. Given that rates of CS delivery are increasing worldwide [2], it is important to ensure that risks to the mother are as minimal as possible; therefore, the results of this study may provide important data to define the future uniform use of vaginal antiseptic wash immediately prior to CS and to determine the best antiseptic wash material in reducing post-operative infections or complications. This study is strengthened by (1) its focus on the investigation of infection rates in complex patient profiles comprising many comorbidities and high BMI rates and (2) the capacity to add data from a regional population since regional data is lacking in the current body of literature. Possible limitations include the study only being conducted at one regional hospital and that the rate of post-CS infection is already low.

## Trial status

Protocol version number: 6

Protocol date: 18 December 2020

Recruitment start date: September 2020

Planned recruitment end date: December 2021

## Abbreviations

AE: Adverse event
CS: Caesarean section
ERA: Enhanced recovery after caesarean delivery
GCP: Good Clinical Practice
m/cs: Microscopic examination, culture and sensitivity
PICF: Participant information and consent form
SSI: Surgical site infection
WHO: World Health Organization
